# Viruses Special Issue “Transfusion-Transmitted Viral Infections”

**DOI:** 10.3390/v15010086

**Published:** 2022-12-28

**Authors:** Daniel Candotti

**Affiliations:** Department of Virology, Henri Mondor Hospital, Paris-Est University, INSERM U955—IMRB—Team 18, 51 Avenue du Maréchal de Lattre de Tassigny, 94010 Créteil, France; daniel.candotti@inserm.fr

Blood transfusion is a life-saving treatment that requires patients to have access to safe and adequate blood products that are available in a timely manner. For decades, the transmission of viral infections through the transfusion of whole blood or blood products has been considered as an unavoidable risk. Any viral infection is likely to be transmitted by transfusion if it has an asymptomatic blood-borne phase and if the infectious virus has the capacity to survive or persist in the collected blood products and cause infection by the intravenous route. The implementation of blood donor selection and the constitution of pools of repeat donors, the increase in our knowledge about viruses, and the unprecedented and continuous development of highly sensitive and specific serologic and molecular screening assays over the past two decades have significantly reduced the risk of transfusion-transmitted viral infections, as reported by Cappy et al., Lelie et al., and Niederhauser et al. [1,2,3] in this Special Issue, entitled “Transfusion-Transmitted Viral Infections”. However, transfusion safety remains a continuous challenge due to changes in the nature and epidemiology of blood-borne viral infections, prompting blood transfusion operators to maintain the surveillance of active infections and/or exposure to the major established viral infections identified in the blood donors (Cappy et al.; Niederhauser et al. [1,3]) and to ensure that the performance of screening assays remains optimal. Monitoring the genotype variability within viral species can be also useful for establishing the most representative international reference materials in order to evaluate and validate screening tests, as suggested by van Drimmelen and Lelie [2,4]. Blood operators must also evaluate the potential threats associated with (re)emerging viral infections in light of the increasing global circulation of human and animal populations, human activities carrying an increased risk of zoonotic infections, and the geographical expansion of viral vectors. In recent years, this has been particularly well-demonstrated by the emergence and spreading of arboviruses (i.e., WNV, CHIKV, ZIKV, JEV) and other zoonoses (i.e., HEV) on a global scale, resulting in the implementation of additional specific and sometimes sporadic screening measures. In addition, the studies of Piron and colleagues and Speicher and colleagues [5,6] remind us that it might be necessary to regularly re-assess the risks associated with long-known viruses such as HTLV-1/2 and HHV-8, even if these infections were not previously considered to represent a priority risk according to the data available at that time. The successfully developed viral metagenomic based on next-generation sequencing has unprecedented potential to identify unknown or poorly characterized viruses in blood donors but is affected by the major limitation of its inability to define the clinical relevance of the identified viruses to blood safety, as reviewed by Slavov and colleagues [7]. Updated epidemiological data are essential, but they must be combined with the accurate monitoring of transfusion transmission by hemovigilance systems, whenever available, so as to decide whether there is a real risk to blood safety. The proper assessment of the residual risk based on validated infectivity models is necessary in order to determine whether risk reduction measures are justified and feasible without threatening blood sufficiency and costs, as outlined by Hoad and colleagues [8] in a study regarding JEV screening in Australia. A good example of the need for a realistic approach to a major new epidemic is the rapid clearance of SARS-CoV2 as a threat to blood safety, despite the presence of the virus in the blood, through studies conducted on blood services. Not only viral variability but also the therapeutic management of viral infections may raise serious concerns regarding blood safety. HIV-infected individuals on antiretroviral therapy (ART) or pre-exposure prophylaxis (PrEP) may volunteer to donate blood without disclosing their HIV status. If these treatments are initiated early enough during the primary infection stage, HIV infection may be undetected due to absence of seroconversion, or even seroreversion, and viral loads below the limit of detection of nucleic acid testing assays. If collected and transfused, such blood can transmit small amounts of potentially infectious virions to the recipient. Vermeulen and colleagues [9] addressed this concern in regard to South African blood donors and concluded that donors on ART may constitute a HIV transmission risk when relying on less sensitive serological rapid tests for screening, as is frequently the case in resource-limited countries. Although the risk appears to be extremely low when using the latest generation of high-throughput serological tests, further studies are required to monitor this emerging risk to blood safety.

The evaluation of the transfusion-transmitted viral residual risk is based on the development and validation of theoretical models established from not only the knowledge of the epidemics and the natural histories of viral infections but also the analytical performances of serological and molecular screening tests and their associations with more or less complex screening algorithms. Lelie and colleagues [2] showed that the efficacy of the method of reducing the risk of HBV transmission through multiple screening strategies of different sensitivities in areas with different levels of endemicity varied considerably. Using the Weusten models and an extensive multi-regional database, they suggested that HBV DNA testing, in the context of individual donation, may be considered as an alternative to nucleic acid testing of mini-pools of plasma and serology-based testing algorithms and that serologic testing should be restricted to first-time donors only. However, this requires that blood banks have the financial resources and infrastructure required to implement such strategies and that the molecular tests used have the appropriate sensitivity. However, screening assays with a high analytical sensitivity may lead to an increased risk of false positive results, leading to donated blood being discarded and permanent donor deferral. Therefore, the further development of complex testing algorithms seems to be essential in order to confirm initially reactive results and, thus, preserve and maintain a stable blood supply, as explained by Deng and colleagues [10] in China, a highly endemic region for HBV. 

The question of research and development (R&D) activities in molecular virology in the case of blood banks and national or regional blood transfusion services is frequently raised. Transfusion operators may limit their activity to the screening of donations and the production and distribution of safe blood products. However, the different studies presented in this Special Issue support the maintenance of R&D activities by blood operators, who are most qualified to define the specific needs in terms of blood safety and the most efficient technical developments required to meet these needs. In addition, blood transfusion services play an important, but often underappreciated, role in general public health, as illustrated by the experience of the Canadian Blood Services reported by Drews, O’Brien, and their colleagues [11,12]. Blood services’ involvement in public health includes support for the surveillance of emerging viral infections beyond blood-borne viruses (i.e., SARS-CoV2) using the blood donor population as a sentinel group (O’Brien et al. [12]), together with the detection and rapid treatment of unsuspected viral infections (van den Berg et al. [13]) and the characterization and development of new therapeutics (Lachert et al. [14]). 

There are still great inequalities around the world regarding transfusion-related viral infectious risks, depending on the combination of local economic and structural resources, the epidemiological situation, and the perception of an acceptable level of residual risk according to the population, regulators, and transfusion operators themselves. It is therefore essential to continue research and assay development in the context of blood transfusion services in order to ensure and improve, when required, optimal viral blood safety. Finally, I would like to thank all the authors and reviewers who contributed to this Special Issue. 
